# Enrichment and fluorogenic labelling of 5-formyluracil in DNA†
†Electronic supplementary information (ESI) available. See DOI: 10.1039/c7sc00637c



**DOI:** 10.1039/c7sc00637c

**Published:** 2017-04-05

**Authors:** Chaoxing Liu, Yafen Wang, Xiong Zhang, Fan Wu, Wei Yang, Guangrong Zou, Qian Yao, Jiaqi Wang, Yuqi Chen, Shaoru Wang, Xiang Zhou

**Affiliations:** a College of Chemistry and Molecular Sciences , Key Laboratory of Biomedical Polymers of Ministry of Education , The Institute for Advanced Studies , Wuhan University , Hubei Province Key Laboratory of Allergy and Immunology , Wuhan , Hubei 430072 , P. R. China . Email: xzhou@whu.edu.cn ; Fax: +86-27-68756663 ; Tel: +86-27-68756663

## Abstract

Recently, the detection of natural thymine modified 5-formyluracil has attracted widespread attention. Herein, we introduce a new insight into designing reagents for both the selective biotin enrichment and fluorogenic labelling of 5-formyluracil in DNA. Biotinylated *o*-phenylenediamine directly tethered to naphthalimide can switch on 5-formyluracil, under physiological conditions, which can then be used in cell imaging after exposure to γ-irradiation. In addition, its labelling property caused the polymerase extension to stop in the 5-formyluracil site, which gave us more information than the fluorescence did itself. The idea of detecting 5-formyluracil might be used in the synthesis of other modified diaminofluoresceins.

## Abstract 2

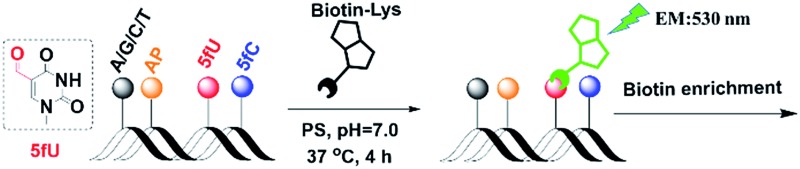
Biotinylated *o*-phenylenediamine directly tethered to naphthalimide can both enrich and fluorogenically label 5-formyluracil in DNA under physiological conditions.

## Introduction

The recent discovery of formylpyrimidines in genomic DNA has energized the field of epigenetics. 5-Formyl-2′-deoxycytidine (5fC) and 5-formyl-2′-deoxyuridine (5fU) have been identified as crucially important forms of canonical nucleoside modifications that play significant roles in gene expression^1^ or are regarded as oxidative lesions that lead to gene regulation, such as introducing mispairing, causing genotoxic lesions, inducing perturbations of DNA function and altering DNA structures.^2^ Methods to sensitively and selectively detect formylpyrimidines have the potential to facilitate an improved understanding of epigenetics. 5fC can be effectively labelled by amine,^3^ hydrazine,^4^ aminoxyl,^5^ indantrione^6^ and indole derivatives.^7^ However, research on highly tagging 5 fU, where 5fC and the abasic sites (AP) cannot disturb the detection of 5fU, has been sparse. Matsuda and co-workers first realized a breakthrough in the highly selective fluorescence “switch-on” of 5fU in a 100 mM NaOH solution with a high signal-to-noise ratio after reacting it with the reagent bis(4,5-dimethoxyanilin-2-yl)disulfide.^8^ However, this reagent is not suitable for the enrichment of 5fU because it can also react with AP, though it cannot disturb the fluorogenic detection of 5fU. In 2015, Höbartner and co-workers reported a remarkable and significant fluorogenic labelling method towards 5-formyluracil in both DNA and RNA at pH 6.0 and 45 °C, for 6 h, by the indole reagent.^7^ However, this also could not be used in imaging 5fU in cells and is not easily modified to enrich 5fU in the genome. Balasubramanian and co-workers explored a biotinylated *o*-phenylenediamine linker that can selectively enrich fragments containing 5fU in DNA by exploiting the chemoselective reactivity of the aldehyde present in 5fU.^9^ The research was timely, systematic and noteworthy. However, when *o*-phenylenediamine reacted with 5fU to form a benzimidazole, there was no fluorescence, except under acidic conditions, according to our previous report.^10^ 5-Hydroxymethyl-2′-deoxyuridine (5hmU) is easily oxidized to form 5fU by KRuO4,^9^ and recent research revealed that 5hmU can be an important epigenetic mark because thymidine (T) can be enzymatically oxidized by TET enzymes to generate the 5hmU form during mouse embryonic stem cell differentiation.^11^ Thus, we wanted to find a reagent that can not only realize the fluorescence “switch-on” of 5fU but also enrich 5fU through linking with a biotin tag that can be used in the streptavidin-coated magnetic bead system. Reagents that can fluorescently label target nucleosides and enrich them have potential applied value in mapping target nucleosides through the nanochannels.^12^ To image 5fU in the cell, the reaction should be under physiological conditions (37 °C, PS buffer and neutral pH value), which provides even further requirements in order for us to design a reagent to meet all of these demands.

**Scheme 1:**
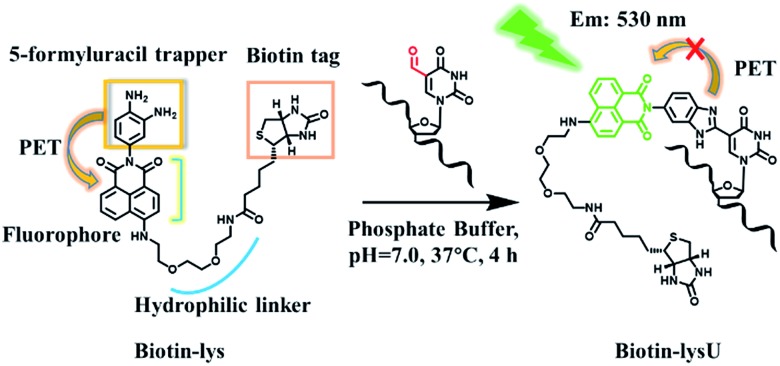
A strategy for the biotin enrichment and fluorogenic labelling of 5-formyluracil in DNA.

Herein, we explored a strategy to solve this problem. In designing a reagent to selectively fluorescently tag 5fU, we observed a series of compounds in which *o*-phenylenediamine was linked to the fluorophore (diaminofluoresceins).^13^ Due to the photoinduced electron transfer (PET) effect, *o*-phenylenediamine turns off the fluorescence of the fluorophores naphthalimide,^14^ BODIPY^15^ and cyanine.^16^ *o*-Phenylenediamine is also an effective trapper of 5fU.^9,10^ Thus, in Scheme 1, *o*-phenylenediamine is designed not only as an electron donor (a fluorescence quencher) for naphthalimide but also as a 5fU trapper that is directly tethered to the imide position of naphthalimide. The polyethylene glycol linker both makes the overall reagent more hydrophilic and allows a greater distance between the biotin and the reactive site.

## Results and discussion

### Evaluating the reactivity of 5-formyluracil with the compound Lyso-NINO

**Fig. 1:**
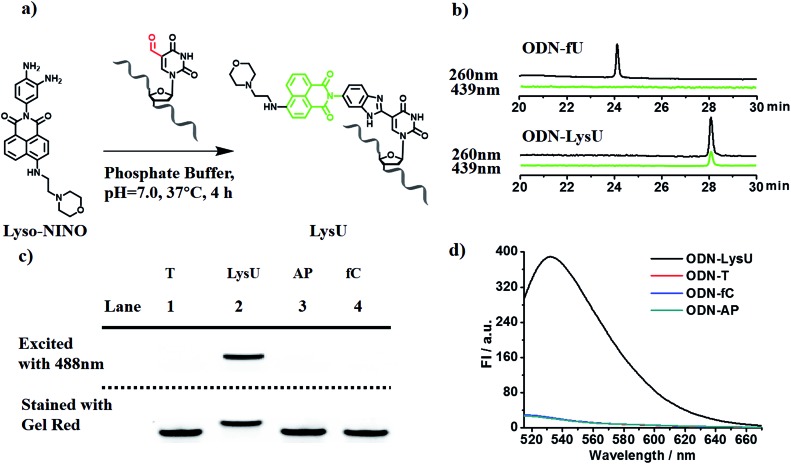
(a) A strategy for the Lyso-NINO fluorogenic labelling of 5-formyluracil in DNA. (b) RP-HPLC trace at *λ* = 260 nm (black) and 439 nm (green) of ODN-5fU and ODN-LysU, which was generated by the reaction with Lyso-NINO under optimized conditions. (c) Polyacrylamide gel electrophoresis analysis of ODN-5fU after incubation with Lyso-NINO (lane 2) before (above dashed line, fluorescence mode, *λ*_ex_: 488 nm) and after (below dashed line, fluorescence mode, *λ*_ex_: 532 nm) being stained with Gel Red in comparison with other DNA controls such as ODN-T (lane 1), ODN-AP (lane 3) and ODN-fC (lane 4) under the same conditions. (d) Fluorescence emission spectra (*λ*_ex_: 439 nm, *λ*_em_: 530 nm) of ODN-5fU after incubation with Lyso-NINO (black line) in comparison with other DNA controls such as ODN-T (red line), ODN-AP (cyan line) and ODN-fC (blue line) under the same conditions.

To explore the feasibility of this approach, we first evaluated the reactivity of 5fU with the compound Lyso-NINO, a two-photon fluorescent probe known to detect endogenous NO in cells,^14^ that had the same *o*-phenylenediamine linked to the naphthalimide structure (Fig. 1a). Lyso-NINO reacted with 5fU in methanol to generate a fluorescent nucleotide named LysU (Fig. S1†). The absorbance and fluorescence emission properties of LysU were investigated in various buffer solutions. The absorbance was detected at 439 nm, and the fluorescence emission maxima were found at 530 nm. (Fig. S15a and S15c†). We then used a 15-mer oligodeoxyribonucleotide ODN-5fU containing one 5fU site as a model reaction test, which was synthesized using an efficiently protected 5-formyluracil phosphoramidite reported recently.^2e,17^ We incubated Lyso-NINO with ODN-5fU under optimized conditions (50 mM PS buffer, pH 7.0, 37 °C, and 4 h). The RP-HPLC (monitored at 260 nm and 439 nm) analysis showed complete conversion to the new product ODN-LysU (Fig. 1b). Lyso-NINO labelled DNA was identified by MALDI-TOF (Fig. S4 and S5†) to ensure the reaction integrity. As for the selectivity, we also treated ODN-T (with the 5fU site replaced by T), ODN-5fC (with the 5fU site replaced by 5fC), and ODN-AP (with the 5fU site replaced by AP) as the negative controls. The RP-HPLC (monitored at 260 nm and 439 nm) analysis showed no reaction in the DNA controls (Fig. S8–S10†). We also attempted to detect different ODNs through denaturing polyacrylamide gel electrophoresis (PAGE) analysis. Due to the good fluorescence properties of ODN-LysU, the fluorogenic labelling DNA band can be directly detected using the Pharos FX Molecular imager (Bio-Rad, USA) (*λ*_ex_: 488 nm), while the others cannot be seen. The gel was then stained with Gel Red to obtain the other DNA bands (*λ*_ex_: 532 nm). The slower migration of ODN-LysU was due to its larger molecular weight (Fig. 1c). The exciting selectivity of the fluorogenic labelling of 5-formyluracil in DNA can also be verified by the fluorescence readout. A dramatic fluorescence enhancement (*λ*_ex_: 439 nm, *λ*_em_: 530 nm) for 5fU can be observed compared to that of other oligodeoxyribonucleotides (ODN-T, ODN-5fC, ODN-AP) (Fig. 1d).

### Examining the status of 5-formyluracil at a particular position on the target DNA

**Fig. 2:**
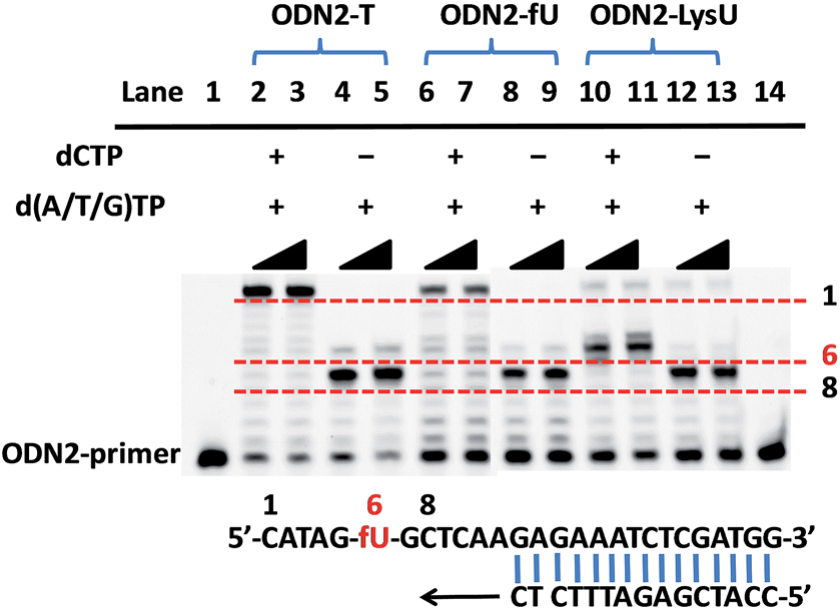
Primer-extension assay with Bsu DNA polymerase. Lane 1, 14: FAM-labeled ODN2-primer as marker; lanes 2, 3, 4, 5: unmodified DNA (ODN2-T); lanes 6, 7, 8, 9: ODN2-5fU; lanes 10, 11, 12, 13: ODN2-LysU (ODN2-5fU after incubation with Lyso-NINO). Lanes 2, 4, 6, 8, 10, 12, reaction time was 1 minute; lanes 3, 5, 7, 9, 11, 13, reaction time was 3 minutes.

To further explore whether the strategy can be used to examine the status of 5fU modification at a particular position on the target DNA, we used ODN2-5fU as a model test using a similar method described by Höbartner *et al.* ^7^ The 5fU site of ODN2-T was replaced by T, whereas the other sites were the same as that in the ODN2-fU sequence; ODN2-LysU and ODN2-fU were then incubated with the reagent Lyso-NINO. The site-specific analysis of ODN2-fU in primer-extension assays showed that the strategy-labelled nucleosides may act as a “roadblock” to abort the primer extension by the Bsu DNA polymerase (NEB) in a reaction time of 1 min or 3 min and enable the detection of 5fU (Fig. 2).

### Evaluating the reactivity of 5-formyluracil with the biotinylated reagent Biotin-Lys

**Fig. 3:**
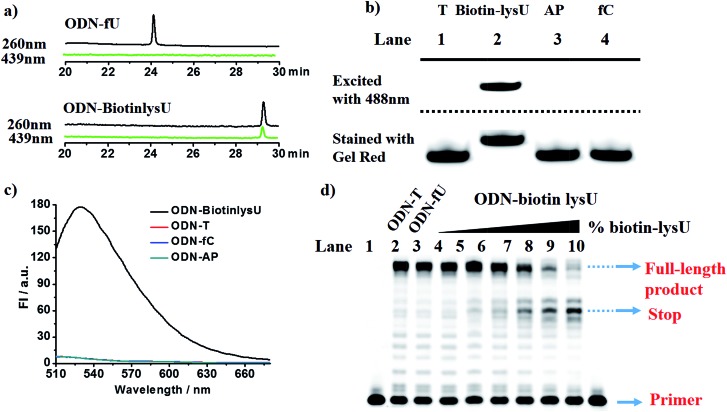
(a) RP-HPLC trace at *λ* = 260 nm (black) and 439 nm (green) of ODN-5fU and ODN–BiotinlysU, which was generated by the reaction with Biotin-lys under optimized conditions. (b) Polyacrylamide gel electrophoresis analysis of ODN-5fU after incubation with Biotin-lys (lane 2) before (above dashed line, fluorescence mode, *λ*_ex_: 488 nm) and after (below dashed line, fluorescence mode, *λ*_ex_: 532 nm) being stained with Gel Red in comparison with other DNA controls such as ODN-T (lane 1), ODN-AP (lane 3) and ODN-fC (lane 4) under the same conditions. (c) Fluorescence emission spectra (*λ*_ex_: 439 nm, *λ*_em_: 530 nm) of ODN-5fU after incubation with Biotin-lys (black line) in comparison with other DNA controls such as ODN-T (red line), ODN-AP (cyan line) and ODN-fC (blue line) under the same conditions. (d) ODN-fU and its unmodified analogue ODN-T were mixed in different fractions (containing 0, 5, 10, 20, 80, 100% 5fU/5fU + T) for a final amount of 2 pmol (from lane 4–9). The mixtures of ODN-fU and ODN-T were incubated with 10 mM Biotin-lys in PS buffer at 37 °C for 4 h. After purification, they were subjected to a primer extension reaction and were finally detected in 20% denaturing PAGE in the presence of 8 M urea.

With these encouraging findings, we commenced synthesizing the biotinylated reagent Biotin-lys (Fig. S2†). The compound can also react with 5fU to generate a fluorescent nucleotide named Biotin-lysU (Fig. S3†). The absorbance and fluorescence emission analysis (Fig. S15b and S15d†) showed that the fluorophore naphthalimide was PET-quenched by the electron rich amino substituents of *o*-phenylenediamine; however, the conversion to the benzimidazole functionality resulted in a turn-on emission because of the blocking of the PET quenching. Next, we treated ODN-5fU with the reagent Biotin-lys under the same conditions as with Lyso-NINO for different times (from 0 to 4 h). From the PAGE analysis results (Fig. S16d†), 4 h is long enough to produce a complete reaction. The RP-HPLC (monitored at 260 nm and 439 nm) analysis showed complete conversion into the new product ODN–biotinlysU (Fig. 3a). The biotin-lys labelled DNA was identified by MALDI-TOF (Fig. S6 and S7†) to ensure the reaction integrity. As for the selectivity, we also treated ODN-T (with the 5fU site replaced by T), ODN-5fC (with the 5fU site replaced by 5fC), and ODN-AP (with the 5fU site replaced by AP) as the negative controls. The RP-HPLC (monitored at 260 nm and 439 nm) analysis showed no reaction in the DNA controls (Fig. S11–S13†). We also attempted to detect different ODNs through PAGE analysis. Due to the good fluorescence properties of the ODN–biotinlysU, the fluorogenic labelling DNA band can be directly detected using the Pharos FX Molecular imager (Bio-Rad, USA) (*λ*_ex_ = 488 nm), while the others cannot be seen. The gel was then stained with Gel Red to obtain the other DNA bands. The slower migration of ODN–biotinlysU was due to its larger molecular weight (Fig. 3b). The exciting selectivity of the fluorogenic labelling of 5fU in DNA can be also verified by the fluorescence readout. A dramatic fluorescence enhancement (*λ*_ex_: 439 nm, *λ*_em_: 530 nm) for 5fU can be observed compared to that of other ODNs (ODN-T, ODN-5fC, ODN-AP) (Fig. 3c). The next step was to determine whether the reagent could be used to obtain 5fU quantitative information on the specific site in the DNA sample, although the fluorescence detection could only supply the overall 5fU quantitative information of the DNA sample *via* fluorescence intensity detection by a fluorescence detector device. Firstly, we verified the possibility of BiotinlysU aborting the primer extension. ODN2-5fU was incubated with Biotin-lys as described before. T and 5fU were bypassed by Bsu DNA polymerases, while BiotinlysU acted as a “roadblock” to abort the primer extension in the reaction times of either 1 min or 3 min (Fig. S16a†). We then generated a model mixture by spiking known amounts (0–100%) of ODN2-5fU into the samples of the unmodified DNA analogue ODN2-T, followed by labelling with Biotin-lys. The Bsu DNA polymerase was used in the next primer extension assay, and the larger amounts of Biotin-lys labelled 5fU sites acted as a stronger pause in the polymerase extension process (Fig. 3d). These results demonstrated that the reagent can have immediate application in identifying the status of the 5fU modification at a particular position on the target DNA.

### Fluorescence images of γ-irradiated Hela cells

**Fig. 4:**
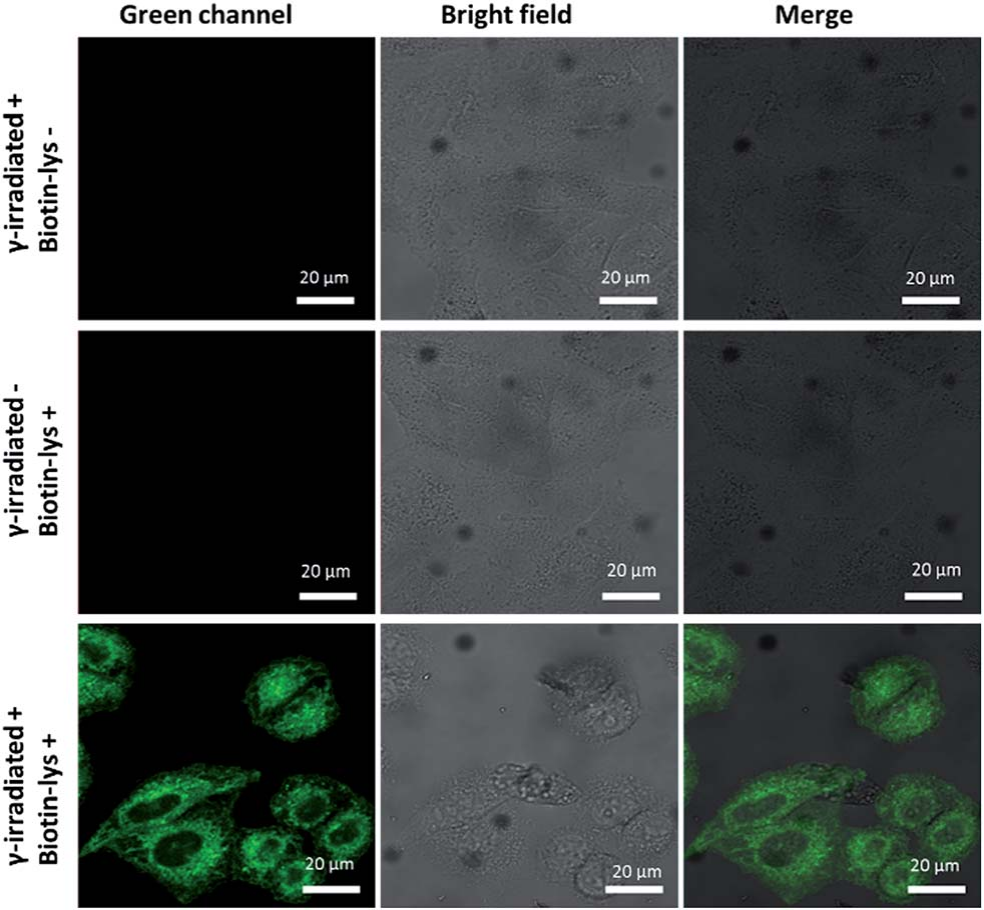
Fluorescence images of Hela cells. Scale bar: 20 μm. The images were acquired with 488 nm excitation and the emission was collected at 500–600 nm.

Pouget and Wang *et al.* reported that different cells exposed to γ-rays would generate 5fU in detectable amounts.^18^ We next exposed Hela cells to ^60^Co irradiation at 1044 Gy (17.4 Gy min^–1^, 60 min) and at room temperature. The unexposed cells were maintained under the same conditions as the negative controls. We then incubated Biotin-lys (10 μM, containing 1% DMSO in DMEM) with the cells at 37 °C for 4 h. Cell imaging was performed on a confocal laser scanning microscope (Nikon C1-si TE2000, Japan) after washing the cells with PBS (0.01 M) three times. The remarkable sensitivity of Biotin-lys was demonstrated by the cell imaging (Fig. 4). Only the cells that were treated with γ-rays, generating 5fU, yielded a notable green fluorescence. To further verify the accuracy of the Biotin-lys reagent reacting with 5fU, we next digested the DNA from the γ-irradiated Hela cells using Degradase Plus (Zymo Research, USA). The LC-MS data showed the exact generation of the Biotin-lysU nucleotide in the DNA from the γ-irradiated Hela cells (Fig. S14†).

Thymidine derivatives like 5hmU and 5fU generally display poor sensitivity when measured by LC-MS/MS, which is likely attributed to their relatively poor proton affinity. Conjugation with the probe may enable the sensitive detection of the modified pyrimidine nucleoside by LC-MS/MS in the future. As a more rigorous consideration, ionizing radiation may produce other reactive aldehydes in DNA that may also conjugate with the probe (*e.g.* certain deoxyribose breakdown products or some DNA adducts arising from by-products of lipid peroxidation). Thus, the fluorescence signal increase observed in Fig. 4 might not be attributed entirely to 5fU in the DNA. More sensitive methods for the *in situ* detection of low abundant 5fU in normal cells or tissues are also needed.

### Enriching 5-formyluracil in DNA fragments

**Fig. 5:**
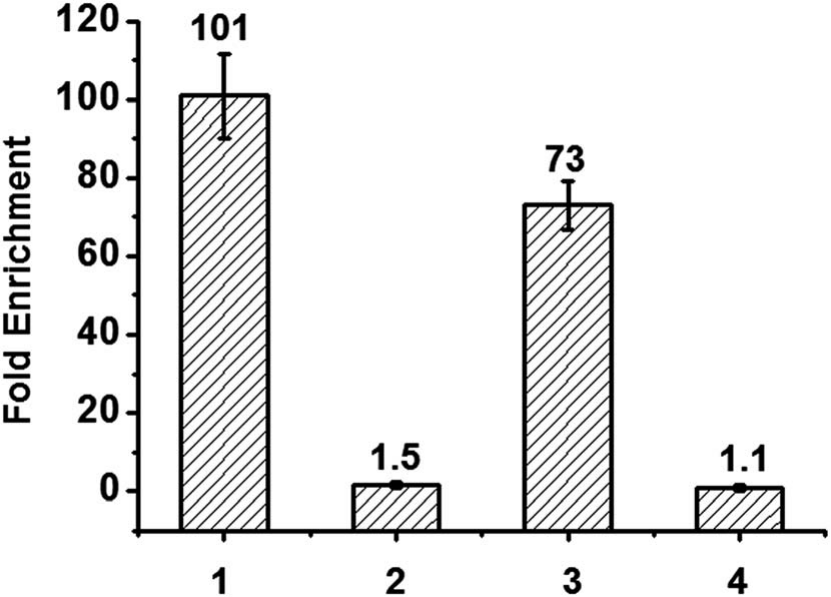
Extent of enrichment of 5-formyluracil in single stranded DNA and double stranded DNA towards 5fC and canonical analogous ODN sequence. Lane 1: ODN-SS-fU/ODN-SS-T; lane 2: ODN-SS-fC/ODN-SS-C; lane 3: ds-DNA-fU/ds-DNA-T; lane 4: ds-DNA-fC/ds-DNA-T.

Finally, we applied the reagent in enriching DNA fragments that contain 5fU. Firstly, we exploited an 80-mer single-stranded ODN bearing two 5fU modifications (ODN-SS-fU) and its canonical analogous ODN sequence (ODN-SS-T) as a control. After incubation with Biotin-lys (50 mM PS buffer, pH 7.0, 37 °C, 6 h), these DNA fragments were enriched by streptavidin-coated magnetic beads. The following qPCR analysis showed a 101-fold enrichment of ODN-SS-fU over ODN-SS-T (Fig. 5 and S17a†), and the PAGE analysis also showed the desirable property of the reagent tagging the 5fU. Only the DNA containing 5fU can react with this reagent (Fig. S16b and S16c†). For the selectivity of 5fC, we also used an 80-mer single-stranded ODN bearing two 5fC modifications (ODN-SS-fC) and its canonical analogous ODN sequence (ODN-SS-C) as a control. Using the same enrichment procedures, the qPCR results showed only a 1.5-fold enrichment of ODN-SS-fC towards ODN-SS-C (Fig. 5, Fig. S17b†). These data demonstrated the effective selective enrichment of 5fU towards its C modification analogue 5fC. We then made a mixture of double-stranded DNA bearing two specific sites per strand (ds-DNA-fU, ds-DNA-fC, and ds-DNA-T), as described by Balasubramanian *et al.* ^9^ We treated it using an optimized pull-down procedure (Fig. S17†). The qPCR results showed a 73-fold enrichment of ds-DNA-fU over ds-DNA-T and a 1.1-fold enrichment of ds-DNA-fC towards ds-DNA-T (Fig. 5 and S17c–e†). The enrichment fold verified the selective capture of 5fU through this reagent. However, the consequence is moderately poorer than that of a biotinylated *o*-phenylenediamine linker (∼150-fold).^9^ In the future, the design of a compound to selectively enrich 5fU in the genome with less steric hindrance and more space between the reactive site and the biotin substitution that can easily be eluted from the streptavidin coated magnetic beads could be considered.

## Conclusions

In conclusion, we created a biotinylated *o*-phenylenediamine directly tethered to naphthalimide that can not only fluorescently tag 5-formyluracil under physiological conditions but also enrich it in DNA. Its remarkable fluorogenic properties made it possible to obtain 5fU quantitative information on the specific site in the DNA sample and imaging cells after their exposure to γ-irradiation. In addition, the strategy for designing a compound to detect 5-formyluracil might be suitable for other diaminofluoresceins.
