# Supplementary material for: Enrichment and fluorogenic labelling of 5-formyluracil in DNA
Source: Chem Sci. 2017 Apr 5;8(6):4505–10. doi: 10.1039/c7sc00637c (PMC5472030; doi:10.1039/c7sc00637c)
Supplement: Supplementary file 1 [file SC-008-C7SC00637C-s001.pdf]

Electronic Supplementary Material (ESI) for Chemical Science.
This journal is © The Royal Society of Chemistry 2017

Supporting Information

# Both enrichment and fluorogenic labelling of 5-formyluracil in DNA

Chaoxing Liu[† ‡], Yafen Wang[† ‡], Xiong Zhang[†], Fan Wu[†], Wei Yang[†], Guangrong Zou[†], Qian Yao[†], Jiaqi Wang[†], Yuqi Chen[†], Shaoru Wang[†], and Xiang Zhou[†]*

Table of Contents

1	General methods and materials	S1
2	Synthesis	S2
3	Table of oligonucleotides sequences	S5
4	DNA MALDI-TOF Mass Spectra	S6
5	HPLC data	S8
6	LC-MS about digesting DNA	S10
7	UV absorption spectra and fluorescent emission spectra	S12
8	Polyacrylamide gel electrophoresis analysis	S13
9	5-formyluracil qPCR enrichment studies	S14
10	Reference	S16

1. General methods and materials

All chemical reagents were purchased from Energy Chemical Co., Ltd. (Shanghai, China) unless mentioned otherwise. HRMS was acquired with Thermo Scientific™ Dionex Ultimate 3000 hybrid LTQ Orbitrap Elite Velos Pro (Thermo Scientific, USA). ^{1}H NMR and ^{13}C NMR spectra were recorded on Bruker Avance 400 NMR spectrometer, respectively. All the canonical oligonucleotides were synthesized and purified by GeneCreate Co., Ltd. (Wuhan, China). And the modification oligonucleotides such as ODN-5fC was bought from Takara Biotechnology (Dalian, China). The ODN-5fU was synthesized using a 5-formyluracil phosphoramidite according to our previous report.[1] ODN-AP was synthesized through ODN-U (one T site was replaced by uracil) treating with Uracil DNA Glycosylase (Invitrogen™, USA). HPLC data was recorded on LC-6AD (Shimadzu, Japan) which equipped with an Inertsil ODS-SP column (5 µm, 250×4.6 mm) (GL Science lnc. Japan) with mobile phase A (100 mM TEAA buffer, pH=7.0) and B (CH_3CN) with a flow rate of 1 mL/min at 35°C (B conc.: 5-5-30% / 0-5-30 min). DNA concentration was quantified by NanoDrop 2000c (Thermo Scientific, USA). DNA MALDI-TOF Mass Spectra were collected on MALDI-TOF-MS (Shimadzu, Japan). Degradase Plus and enzyme reaction buffer were purchased from Zymo Research (Zymo Research, USA). Gel Imaging was monitored with Pharos FX Molecular imager (Bio-Rad, USA). UV absorption spectra were recorded on UV-2550 (Shimadzu, Japan). Fluorescent emission spectra were acquired with PerkinElmer LS 55 (PerkinElmer, USA). LC-MS data were collected with the Agilent™ 1220 Infinity LC combined with the 6120 Single Quadrupole mass spectrometer (Agilent Technologies). pH was measured with Mettler Toledo, FE20-Five Easy™ pH (Mettler Toledo, Switzerland). TLC plates were monitored with portable UV-LAMP (GL-9406, Jiangsu, China). qPCRs were performed using a CFX-96 RealTime System (Bio-Rad, USA).

ODN reaction protocol. ODNs (100 µM, 2 µL), reagents (10 mM in DMSO, 2 µL), PS buffer (1 M, pH=7.0, 5 µL) and 91 µL ddH_2O were added together into 1.5 mL microcentrifuge tube at 37°C for 4 h, respectively.

HPLC analysis of ODNs. ODNS were reacted with reagents through the ODN reaction protocol, then HPLC data was recorded on LC-6AD (Shimadzu, Japan) which equipped with an Inertsil ODS-SP column (5 µm, 250×4.6 mm) (GL Science lnc. Japan) with mobile phase A (100 mM TEAA buffer, pH=7.0) and B (CH_3CN) with a flow rate of 1 mL/min at 35°C (B conc.: 5-5-30% / 0-5-30 min).

Enzymatic digest of ODNs protocol. DNAs in the presence of Degradase Plus (1 µL) and 10×Degradase Plus reaction buffer (2.5 µL) (Zymo Research) in a final volume of 25 µL was digested to its constituent nucleosides via incubation at 37°C for 2 h. The nucleoside mixture was filtered by an ultrafiltration tube (10 kDa cutoff, Amicon, Millipore) to remove the enzymes.

Denaturing polyacrylamide gel electrophoresis (PAGE) analysis. The concentration of each oligonucleotide was 1 µM in 10 µL 80% deionized formamide. A 20% denaturing PAGE was prepared by using 1xTBE buffer (89 mM Boric acid, 2 mM EDTA, 89 mM Tris base) containing 7 M urea. The PAGE was carried out in 1xTBE buffer at a constant voltage of 150 V for about 1 h at room temperature. We scanned the final polyacrylamide gel electrophoresis products with Pharos FX Molecular imager operated in the fluorescence mode (λ_{ex}=488 nm). Then the gel was stained with Gel Red to get other DNA bands (λ_{ex}=532 nm).

The site-specific analysis of ODN2-fU in primer-extension assays. The extension reaction was performed at 37°C for 1 min or 3 min in 1x PCR buffer using 1 µM 5'-FAM-labeled primer (ODN2-Primer), 1 µM DNA template (ODN2-T, ODN2-fU, ODN2-fU after reaction), 200 µM dNTP and 0.05 U/µl BSU DNA polymerase (NEB). Then the primer extensions were analyzed by denaturing PAGE.

2. Synthesis

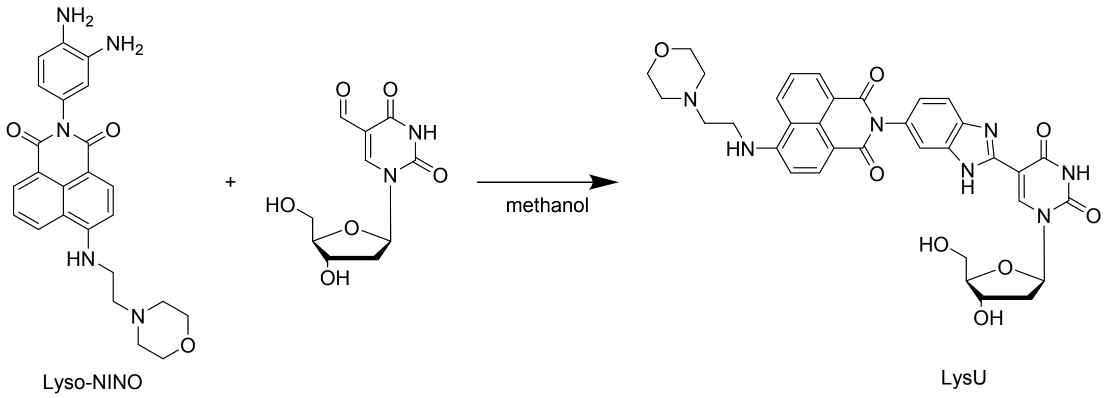

Figure S1. Synthesis of compound LysU.

Synthesis of compound LysU

Lyso-NINO was synthesized according to previous report.[2] Lyso-NINO (86 mg, 0.2 mmol) and 5-formyl-2'-deoxyuridine (51 mg, 0.2 mmol) [3] were dissolved into 50 mL methanol. The reaction mixture was kept stirring at 50°C overnight. The residue was dried in vacuo. The product LysU was purified by silica gel column chromatography using eluent CH_2Cl_2/MeOH (30:1) to yield 107 mg (80%) as a yellow powder. ^{1}H NMR (400 MHz, DMSO-d6) δ = 12.38 (d, *J*=14.0, 1H), 11.99 (s, 1H), 8.88 (d, *J* = 4.5, 1H), 8.70 (d, *J* = 8.5, 1H), 8.70 (d, *J* = 8.5, 1H), 8.45 (d, *J*=7.1, 1H), 8.28 (d, *J* = 8.5, 1H), 7.81 – 7.57 (m, 3 H), 7.49 (s, 1H), 7.05 (dd, *J* = 8.4, 1.8, 1H), 6.85 (d, *J* = 8.8, 1H), 6.22 (d, *J* = 4.8, 1H), 5.36 (dd, *J* = 7.9, 4.0, 1H), 5.24 – 5.00 (m, 1H), 4.30 (s, 1H), 3.90 (s, 1H), 3.63 – 3.54 (m, 8H), 2.70 (d, *J* = 6.0, 2H), 2.51 – 2.50 (m, 2H), 2.26 (s, 2H), 1.28 – 1.15 (m, 2H). ^{13}C NMR (100 MHz, DMSO-d6) δ = 164.71, 163.90, 162.24, 150.90, 149.88, 147.42, 141.57, 134.62, 131.16, 130.75, 130.23, 128.89, 124.75, 123.31, 122.85, 120.62, 118.97, 118.01, 113.33, 112.29, 108.63, 104.32, 104.19, 88.24, 85.82, 70.86, 66.52, 61.70, 56.49, 53.68, 46.10, 40.49. HRMS $C_{34}H_{34}N_7O_8^+$ $[M+H]^+$ calculated 668.24634, found 668.24610.

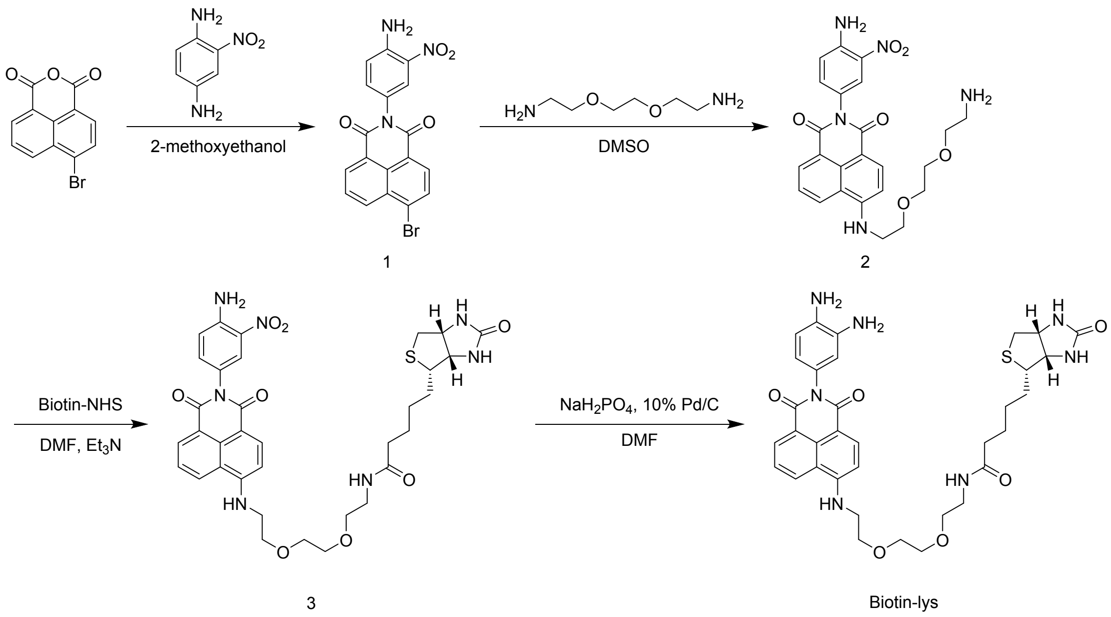

Figure S2. Synthesis of compound Biotin-lys.

Synthesis of compound 2

1 was synthesized according to previous report. [2] 1 (2 g, 4.9 mmol) was dissolved in 30 mL DMSO, then 2,2'-(Ethylenedioxy)bis(ethylamine) (6 mL, 41 mmol) was added. After stirring at 90°C for 5 h, the solution was poured into 500 mL ice. The precipitate was isolated by filtration, further purification through silica gel column chromatography using eluent CH_2Cl_2/MeOH/Et_3N (40:2:1%). Compound 2 was obtained as a yellow solid (2.1 g, 90%). ^{1}H NMR (400 MHz, DMSO-d6) δ = 8.70 (d, *J* = 8.4 Hz, 1H), 8.41 (d, *J* = 7.2 Hz, 1H), 8.23 (d, *J* = 8.6 Hz, 1H), 7.97 (d, *J* = 2.3 Hz, 1H), 7.85 (s, 1H), 7.69 (d, *J* = 7.8 Hz, 1H), 7.65 (d, *J* = 7.0 Hz, 2H), 7.38 (dd, *J* = 8.9, 2.3 Hz, 1H), 7.13 (d, *J* = 9.0 Hz, 1H), 6.83 (d, *J* = 8.7 Hz, 1H), 3.75 (t, *J* = 5.5 Hz, 2H), 3.62 – 3.60 (m, 4H), 3.55 – 3.53 (m, 2H), 3.36 (t, *J* = 5.8 Hz, 2H), 2.64 (t, *J* = 5.8 Hz, 2H), 2.56 – 2.45 (m, 2H). ^{13}C NMR (101 MHz, DMSO-d6) δ = 164.61, 163.69, 151.19, 146.30, 137.52, 134.68, 131.25, 130.27, 130.13, 129.20, 126.22, 124.70, 124.32, 122.65, 120.62, 119.68, 108.37, 104.38, 73.31, 70.32, 70.05, 68.61, 43.24, 41.69. HRMS $C_{24}H_{26}N_5O_6^+$ $[M+H]^+$ calculated 480.18776, found 480.18675.

Synthesis of compound 3

Biotin-NHS (341 mg, 1 mmol), compound 2 (527 mg, 1.1 mmol) were dissolved in 15 mL DMF. 3 drops of Et_3N were added as catalyst. The solution was kept stirring at 50°C for 1 h. Then it was poured into 150 mL ether. The precipitate was isolated by filtration, further purification through silica gel column chromatography using eluent CH_2Cl_2/MeOH (from 40:1 to 15:1). Compound 3 was obtained as a yellow solid (620 mg, 80%). ^{1}H NMR (400 MHz, DMSO-d6) δ = 8.73 (d, *J* = 8.5 Hz, 1H), 8.42 (d, *J* = 7.1 Hz, 1H), 8.25 (d, *J* = 8.5 Hz, 1H), 7.97 (d, *J* = 2.3 Hz, 1H), 7.86 (t, *J* = 5.4 Hz, 2H), 7.74 – 7.66 (m, 1H), 7.64 (s, 2H), 7.37 (dd, *J* = 8.9, 2.3 Hz, 1H), 7.13 (d, *J* = 9.0 Hz, 1H), 6.84 (d, *J* = 8.8 Hz, 1H), 6.46 (s, 1H), 6.41 (s, 1H), 4.34 – 4.26 (m, 1H), 4.15 – 4.08 (m, 1H), 3.75 (t, *J* = 5.6 Hz, 2H), 3.66 – 3.58 (m, 4H), 3.55 (dd, *J* = 5.4, 3.1 Hz, 2H), 3.41 – 3.39 (m, 1H), 3.18 (q, *J* = 5.7 Hz, 2H), 3.09 – 3.04 (m, 1H), 2.81 (dd, *J* = 12.4, 5.0 Hz, 1H), 2.59 (t, *J* = 8.6 Hz, 1H), 2.54 – 2.50 (m, 1H), 2.06 (t, *J* = 7.4 Hz, 2H), 1.58 – 1.49 (m, 4H), 1.28 – 1.19 (m, 2H). ^{13}C NMR (101 MHz, DMSO-d6) δ = 172.64, 164.63, 163.71, 163.22, 151.20, 146.30, 137.51, 134.72, 131.29, 130.30, 130.13, 129.23, 126.21, 124.75, 124.32, 122.69, 120.65, 119.69, 108.41, 104.40, 70.28, 70.04, 69.63, 68.60, 61.49, 59.65, 55.91, 45.98, 43.23, 38.86, 35.55, 28.66, 28.49, 25.73. HRMS $C_{34}H_{40}N_7O_8S^+$ $[M+H]^+$ calculated 706.26536, found 706.26366

Synthesis of compound Biotin-lys

$NaH_2PO_4 \cdot H_2O$ (442 mg, 14 eq.) was dissolved into 4 mL ddH_2O in Schlenk flask under Ar atmospheric environment. Compound **3** (210 mg, 0.30 mmol, 1 eq.) and 56 mg 10% Pd/C were dispersed in 15 mL DMF. After 10 minutes, the DMF solution was added dropwise into Schlenk flask. Then, the reaction mixture was kept violent stirring at 55°C for 2 h under Ar atmosphere. After that, the reaction mixture was poured into 150 mL cool acetonitrile. The precipitate was isolated by filtration, further purification through silica gel column chromatography using eluent CH_2Cl_2/MeOH/Et_3N (30:1:1%). The compound Biotin-lys was obtained 160 mg (80%) as a yellow solid. ^{1}H NMR (400 MHz, DMSO-d6) δ = 8.72 (d, *J*=8.2, 1H), 8.42 (d, *J*=6.9, 1H), 8.25 (d, *J*=8.5, 1H), 7.86 (t, *J* = 5.6, 1H), 7.79 (t, *J* = 5.4, 1H), 7.73

– 7.67 (m, 1H), 6.85 (d, *J* = 8.7, 1H), 6.57 (d, *J* = 8.1, 1H), 6.45 (s, 1H), 6.39 (s, 1H), 6.36 (d, *J* = 2.3, 1H), 6.26 (dd, *J* = 8.1, 2.3, 1H), 4.60 (s, 4H), 4.31 – 4.27 (m, 1H), 4.13 – 4.07 (m, 1H), 3.74 (t, *J*=5.6, 2H), 3.61 – 3.59 (m, 4H), 3.54 (dd, *J* = 5.7, 3.2, 2H), 3.41 – 3.39 (m, 1H), 3.18 (q, *J*=5.8, 2H), 3.05 (dt, *J* = 8.7, 5.9, 2H), 2.80 (dd, *J* = 12.4, 5.1, 1H), 2.57 (d, *J* = 12.4, 1H), 2.53 – 2.49 (m, 1H), 2.05 (t, *J* = 7.4, 2H), 1.58 – 1.47 (m, 4H), 1.26 – 1.15 (m, 2H). ^{13}C NMR (101 MHz, DMSO-d6) δ = 172.65, 164.69, 163.93, 163.21, 150.92, 135.57, 135.09, 134.56, 131.17, 130.16, 128.94, 126.22, 124.82, 122.98, 120.66, 117.78, 115.29, 114.41, 108.81, 104.37, 70.26, 70.03, 69.62, 68.60, 61.48, 59.63, 55.90, 46.03, 43.21, 38.87, 35.55, 28.65, 28.48, 25.72. HRMS $C_{34}H_{42}N_7O_6S^+$ [M+H]$^+$ calculated 676.29118, found 676.28836. $C_{34}H_{41}N_7NaO_6S^+$ [M+Na]$^+$ calculated 698.27312, found 698.27012.

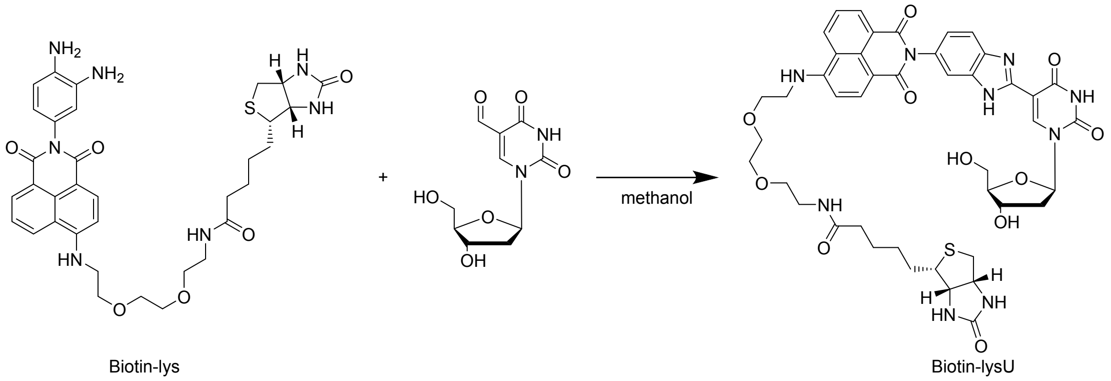

Figure S3. Synthesis of compound Biotin-lysU.

Synthesis of compound Biotin-lysU

Biotin-lys (68 mg, 0.1 mmol) and 5-formyl-2'-deoxyuridine (26 mg, 0.1 mmol) were dissolved into 30 mL methanol. The reaction mixture was kept stirring at 50°C overnight. The residue was dried in vacuo. The product Biotin-lysU was purified by silica gel column chromatography using eluent CH_2Cl_2/MeOH/Et_3N (30:1:1%) to yield 77 mg (85%) as a yellow powder. ^{1}H NMR (400 MHz, DMSO-d6) δ = 12.37 (d, *J* = 15.1, 1H), 8.88 (d, *J* = 4.9, 1H), 8.76 (d, *J* = 8.2, 1H), 8.46 (d, *J* = 7.1, 1H), 8.28 (d, *J* = 8.5, 1H), 7.85 (t, *J* = 5.1, 2H), 7.73 (t, *J* = 7.6, 1H), 7.65 (dd, *J* = 14.6, 8.5, 1H), 7.49 (s, 1H), 7.05 (d, *J* = 8.4, 1H), 6.89 (d, *J* = 8.6, 1H), 6.43 (s, 1H), 6.38 (s, 1H), 6.22 (dd, *J* = 12.0, 6.3, 1H), 5.39 – 5.31 (m, 1H), 5.15 – 5.04 (m, 1H), 4.36 – 4.20 (m, 2H), 4.18 – 4.03 (m, 1H), 3.89 (dd, *J* = 6.1, 2.8, 1H), 3.75 (t, *J* = 5.4, 2H), 3.61 (m, 6H), 3.54 (d, *J* = 4.6, 2H), 3.21 – 3.13 (m, 2H), 3.09 – 3.00 (m, 2H), 2.85 (m, 2H), 2.79 (dd, *J*=12.5, 5.0, 1H), 2.56 (d, *J*=12.5, 1H), 2.26 (d, *J*=4.8, 2H), 2.05 (t, *J*=7.3, 2H), 1.64 – 1.14 (m, 6H). ^{13}C NMR (101 MHz, DMSO-d6) δ = 172.65, 164.83, 164.01, 163.21, 162.29, 151.15, 149.95, 147.35, 134.71, 131.30, 130.37, 129.21, 124.85, 122.92, 120.72, 108.67, 104.44, 88.36, 85.97, 70.93, 70.28, 70.04, 69.62, 68.60, 61.74, 61.48, 59.64, 55.90, 45.92, 43.24, 38.87, 35.55, 28.66, 28.49, 25.73. HRMS $C_{44}H_{50}N_9O_{11}S^+$ [M+H]$^+$ calculated 912.33450, found 912.32979.

3. Table of oligonucleotides sequences

**Oligomer**	**Sequence (from 5'to 3')**
ODN-T	GACTCAA**T**AGCCGTA
ODN-AP	GACTCAA**AP**AGCCGTA
ODN-5fU	GACTCAA**5fU**AGCCGTA
ODN-5fC	GACTCAA**5fC**AGCCGTA
ODN2-T	CATAG**T**GCTCAAGAGAAATCTCGATGG
ODN2-5fU	CATAG**5fU**GCTCAAGAGAAATCTCGATGG
ODN-SS-fU	TCCTCGGCGGTGTTGCTCTCTGTTGTGCCTCCGCCCG**5fU**CA<br>GGCAG**5fU**GGGCAGGACAAGGACGCAGAGCCACAGCCAAGAA
ODN-SS-T	TCCTCGGCGGTGTTGCTCTCTGTTGTGCCTCCGCCCG**T**CA<br>GGCAG**T**GGGCAGGACAAGGACGCAGAGCCACAGCCAAGAA
ODN-SS-fC	CCTATCATCTTATATCTACTACTACTACCTTTAA**5fC**TAAGA<br>TT**5fC**ATGTATAGAATAGATTTAGAGGATTTAGTAGATTTAG
ODN-SS-C	CCTATCATCTTATATCTACTACTACTACCTTTAA**C**TAAGA<br>TT**C**ATGTATAGAATAGATTTAGAGGATTTAGTAGATTTAG
ds-DNA-fU	a) TCCTCGGCGGTGTTGCTCTCTGTTGTGCCTCCGCCCG**5fU**CA<br>GGCAG**5fU**GGGCAGGACAAGGACGCAGAGCCACAGCCAAGAA<br>b) TTCTTGGCTGTGGCTCTGCGTCCTTGTCCTGCCCAC**5fU**GCC**5fU**<br>GACGGGCGGAGGCACAACAGAGAGCAACACCGCCGAGGA
ds-DNA-fC	a) CCTATCATCTTATATCTACTACTACTACCTTTAA**5fC**TAAGA<br>TT**5fC**ATGTATAGAATAGATTTAGAGGATTTAGTAGATTTAG<br>b) CTAAATCTACTAAATCCTCTAAATCTATTCTATA**5fC**ATGAAT**5fC**<br>TTAGTTAAAGGTAGTAGTAGTAGATATAAGATGATAGG
ds-DNA-T	a) GCTCGCTTTGTTGGTTTCCTTGTTCTCTGTGCCCACTGCCTG<br>ACGGGCGGAAAGCAGCGCGAGCAAGCGAGACAGGACAC<br>b) GTGTCCTGTCTCGCTTGCTCGCGCTGCTTTCCGCCCGTCAG<br>GCAGTGGGCACAGAGAACAAGG AAACCAACAAAGCGAGC
Forward Primer 1	TTCTTGGCTGTGGCTCTGCGTCCTTGTCCT
Reverse Primer 1	TCCTCGGCGGTGTTGCTCTCTGTTGTGCCT
Forward Primer 2	CTAAATCTACTAAATCCTCTAAATCTATTC
Reverse Primer 2	CCTATCATCTTATATCTACTACTACTACCT
Forward Primer 3	GCTCGCTTTGTTGGTTTCCTTGTTCTCTGT
Reverse Primer 3	GTGTCCTGTCTCGCTTGCTCGCGCTGCTTT
ODN2- Primer	5'-FAM-CCATCGAGATTTCTC

4. DNA MALDI-TOF Mass Spectra

5'-Phos-GACTCAA**5fU**AGCCGTA-3' ⟶ 5'-Phos-GACTCAA**LysU**AGCCGTA-3'

calculated 5066.2, found 5063.3.

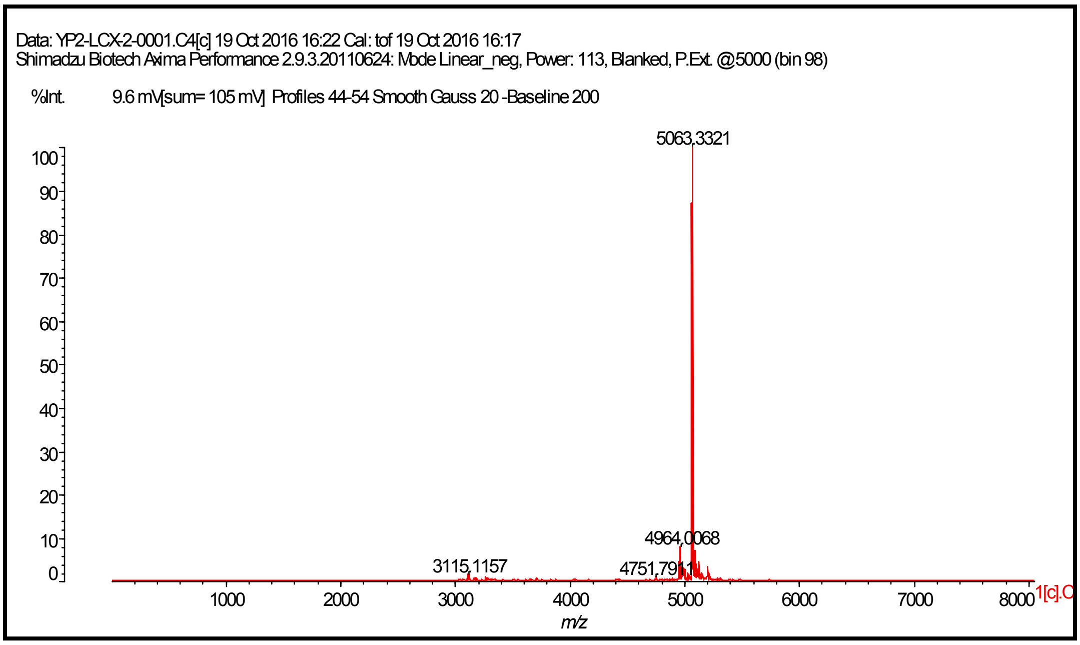

Figure S4. MALDI-TOF-spectrum of ODN-LysU.

5'-CATAG**5fU**GCTCAAGAGAAATCTCGATGG-3' ⟶ 5'-CATAG**LysU**GCTCAAGAGAAATCTCGATGG-3'

calculated 8757.7, found 8758.1.

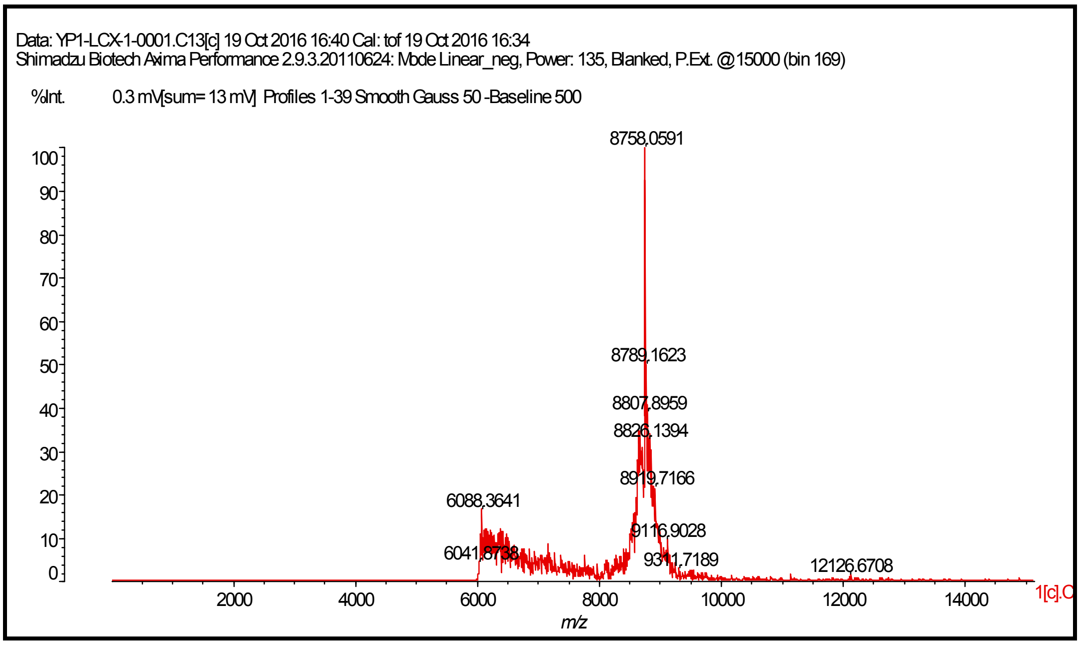

Figure S5. MALDI-TOF-spectrum of ODN2-LysU.

5’-GACTCAA**5fU**AGCCGTA-3’ ⟶ 5’-Phos-GACTCAA**BiotinlysU**AGCCGTA-3’

calculated 5230.3, found 5230.8.

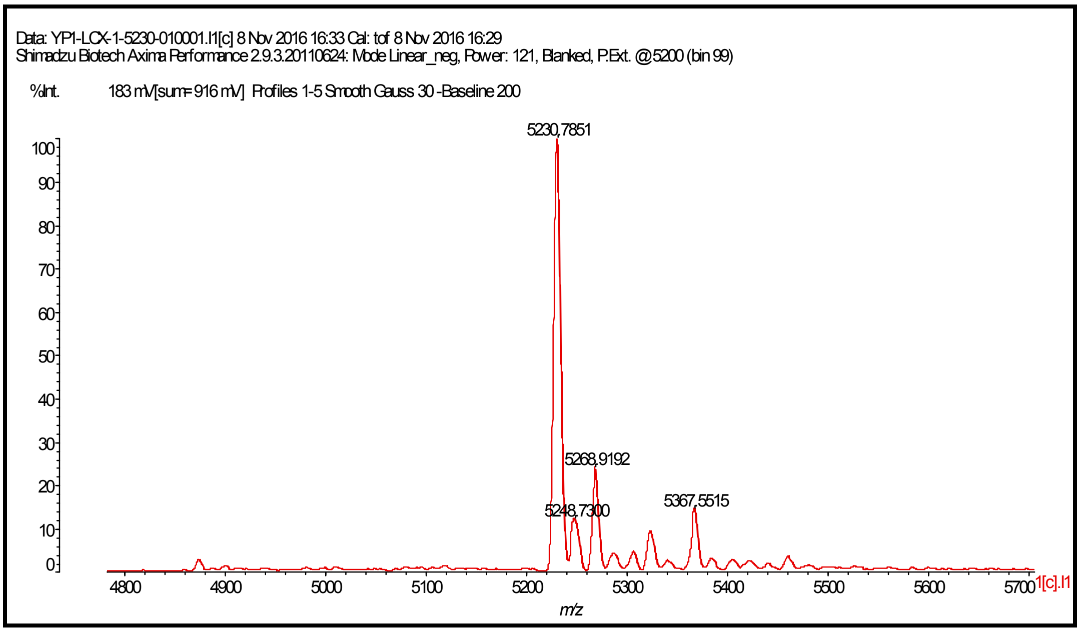

Figure S6. MALDI-TOF-spectrum of ODN-BiotinlysU.

5’-CATAG**5fU**GCTCAAGAGAAATCTCGATGG-3’ ⟶ 5’-CATAG**BiotinlysU**GCTCAAGAGAAATCTCGATGG-3’

calculated 8976.7, found 8975.5.

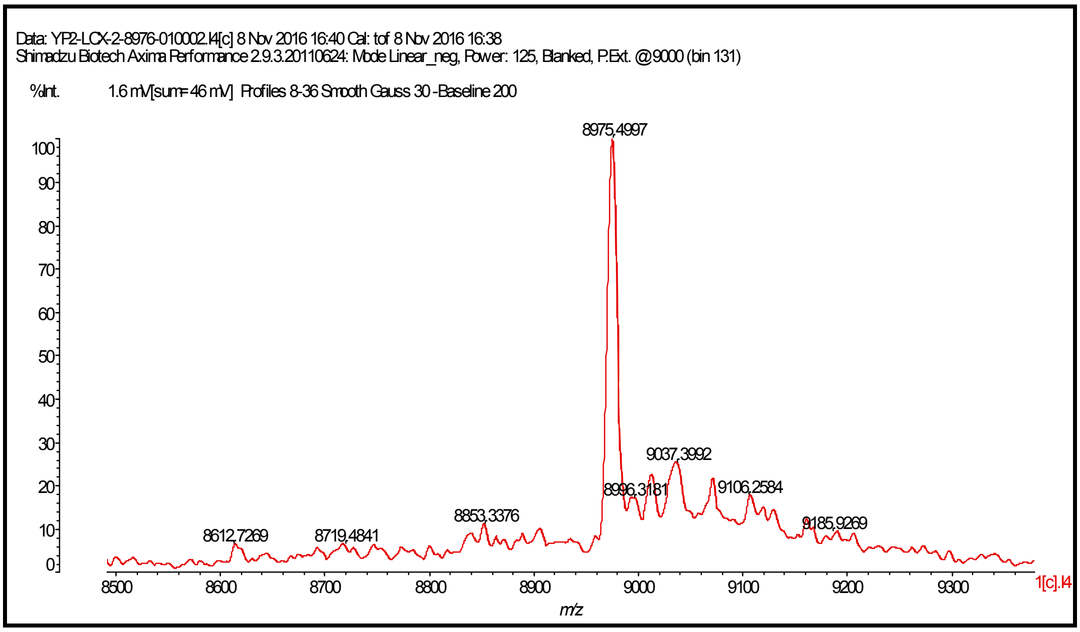

Figure S7. MALDI-TOF-spectrum of ODN2-BiotinlysU.

5. HPLC data

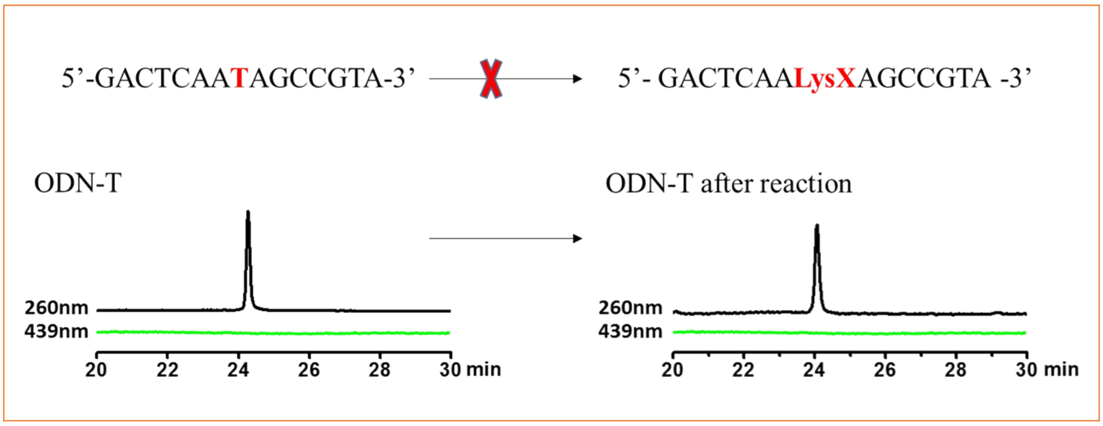

Figure S8. HPLC spectrum about ODN-T and ODN-T after incubation with Lyso-NINO under the ODN reaction protocol.

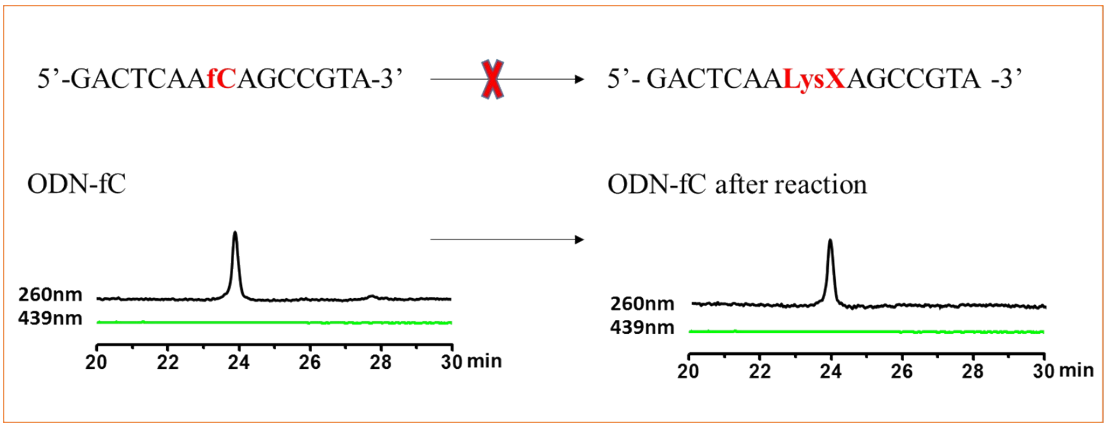

Figure S9. HPLC spectrum about ODN-fC and ODN-fC after incubation with Lyso-NINO under the ODN reaction protocol.

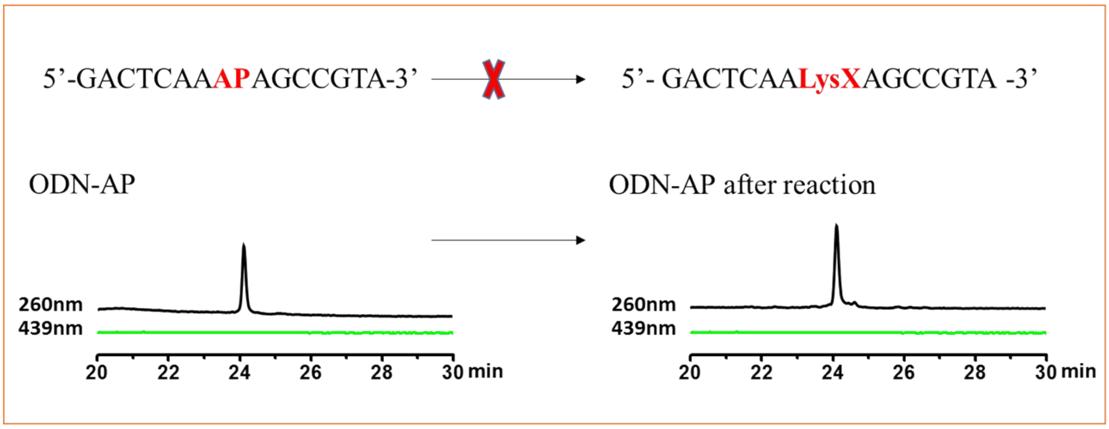

Figure S10. HPLC spectrum about ODN-AP and ODN-AP after incubation with Lyso-NINO under the ODN reaction protocol.

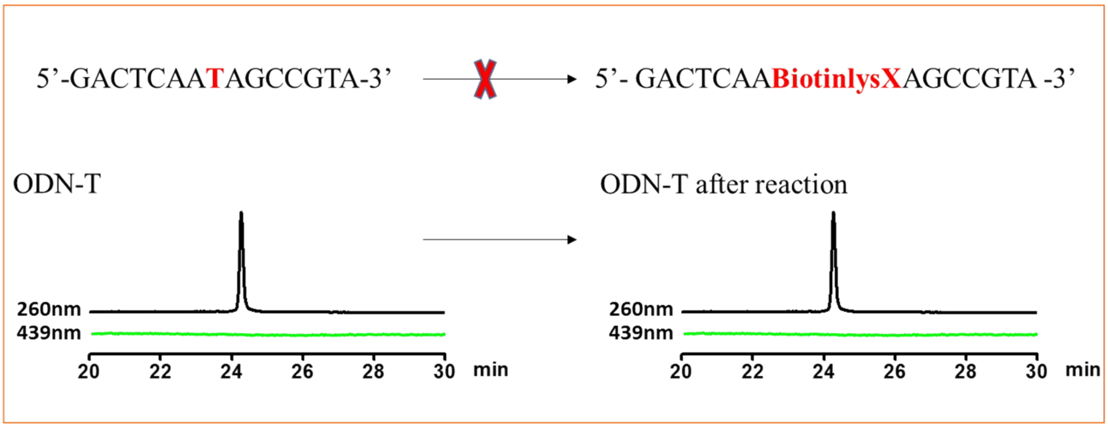

Figure S11. HPLC spectrum about ODN-T and ODN-T after incubation with Biotin-lys under the ODN reaction protocol.

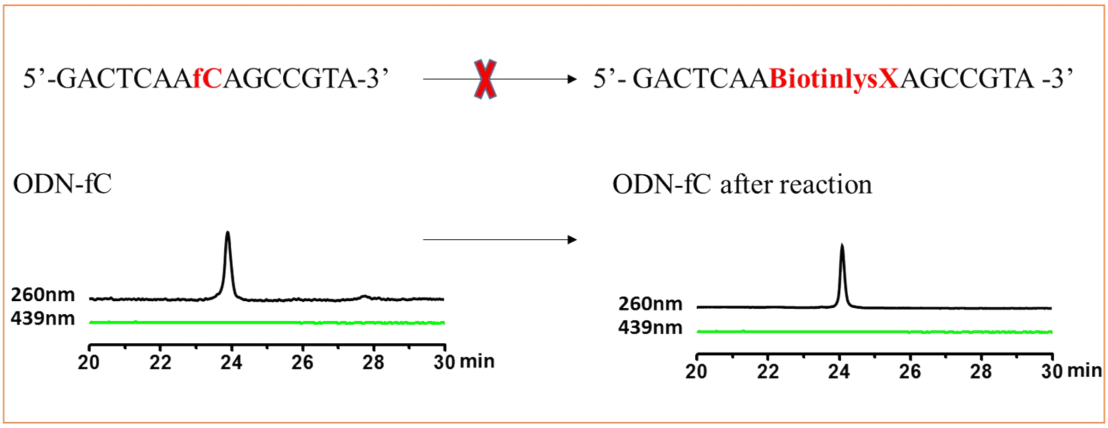

Figure S12. HPLC spectrum about ODN-fC and ODN-fC after incubation with Biotin-lys under the ODN reaction protocol.

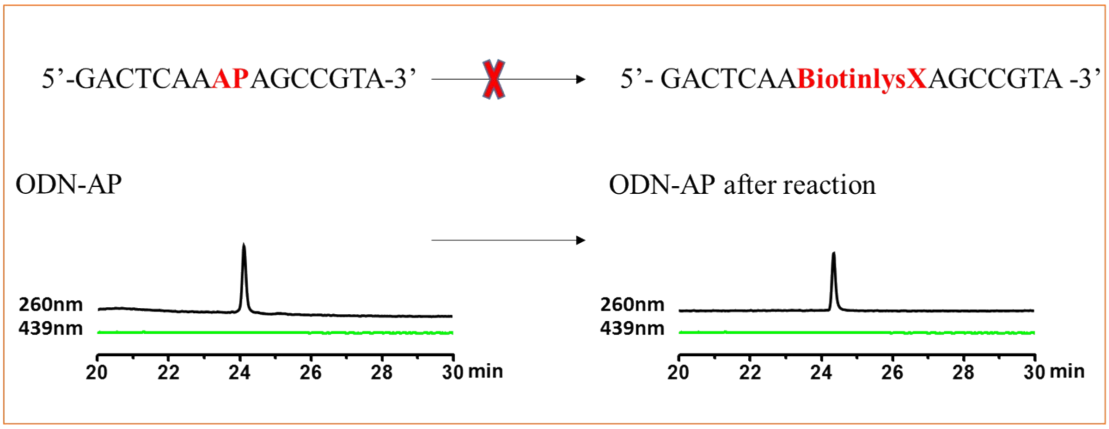

Figure S13. HPLC spectrum about ODN-AP and ODN-AP after incubation with Biotin-lys under the ODN reaction protocol.

6. LC-MS about digest DNA

a)

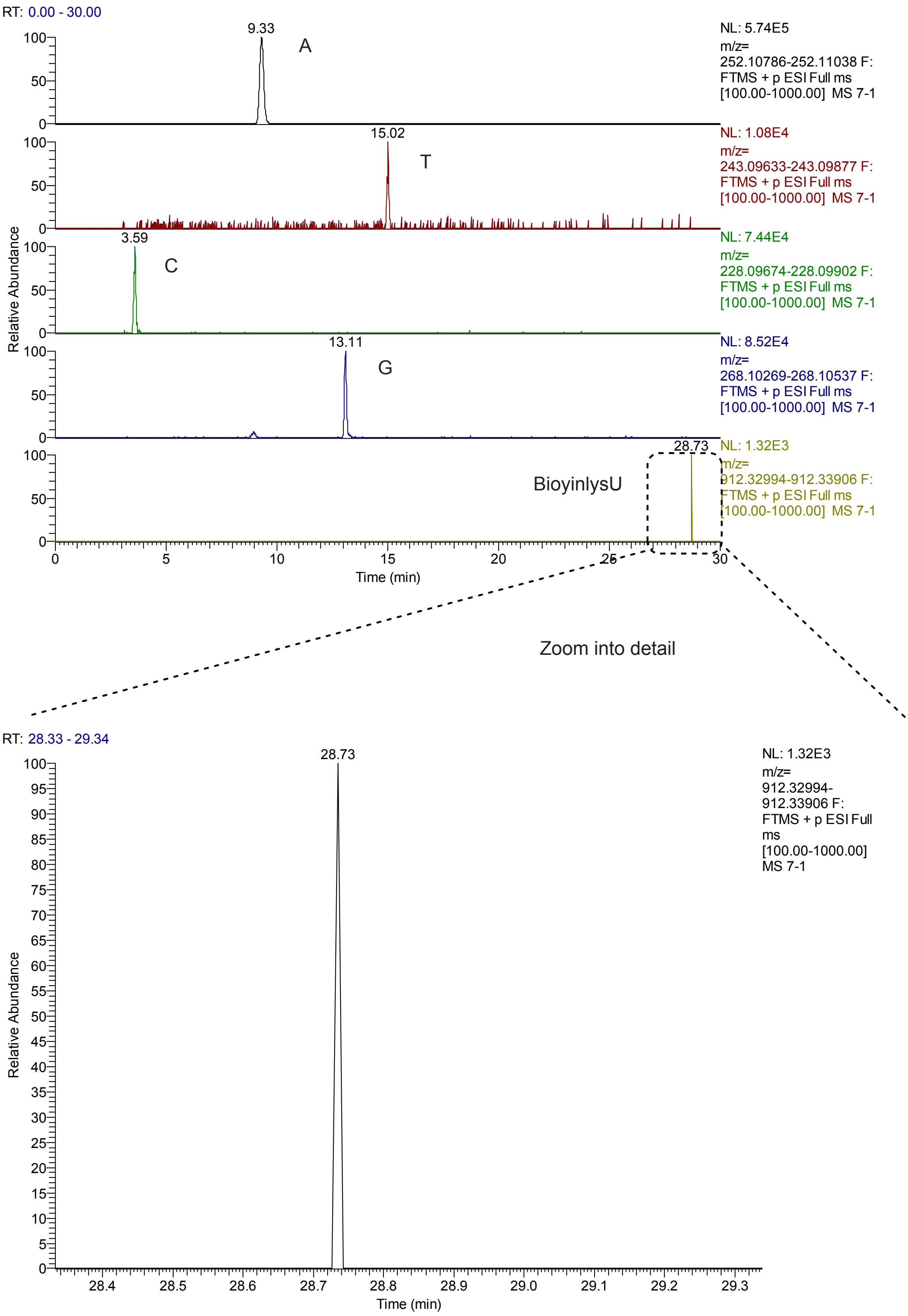

b)

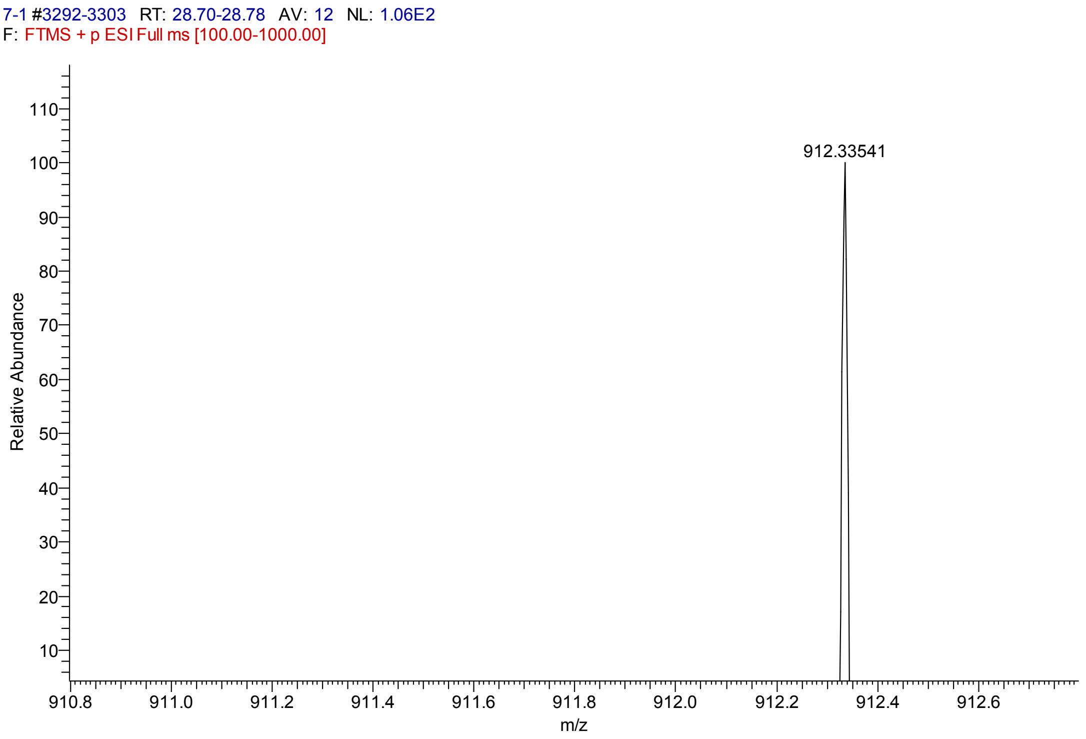

C)

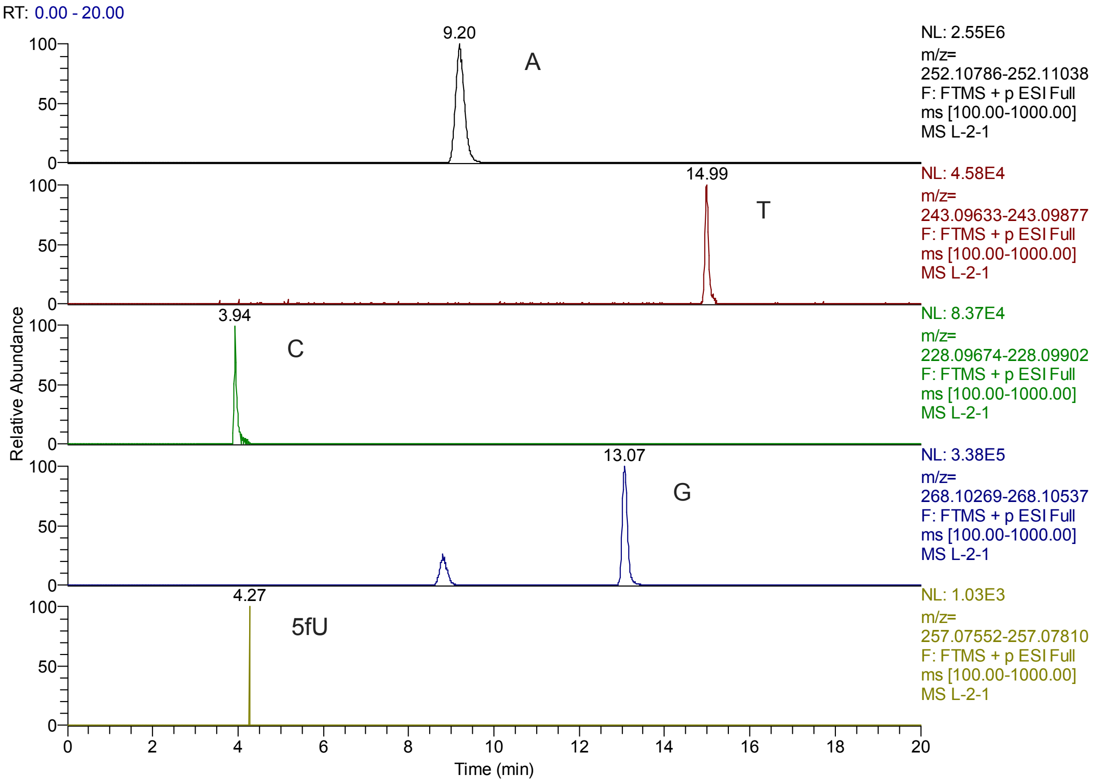

Figure S14. a) HPLC-MS extracted [M⁺H]⁺ ion count for A, T, C, G, Biotin-lysU deoxynucleosides after digestion of DNA from the γ-irradiated Hela cells which was after labeled by Biotin-lys. b) HRMS (ESI+) of Biotin-lysU in HPLC-MS after digestion, HRMS $C_{44}H_{50}N_9O_{11}S^+$ $[M+H]^+$ calculated 912.33450, found 912.33541. C) HPLC-MS extracted $[M^+H]^+$ ion count

for A, T, C, G, 5fU deoxynucleosides after digestion of DNA from the γ-irradiated Hela cells which was before labeled by Biotin-lys.

7. UV absorption spectra and fluorescent emission spectra

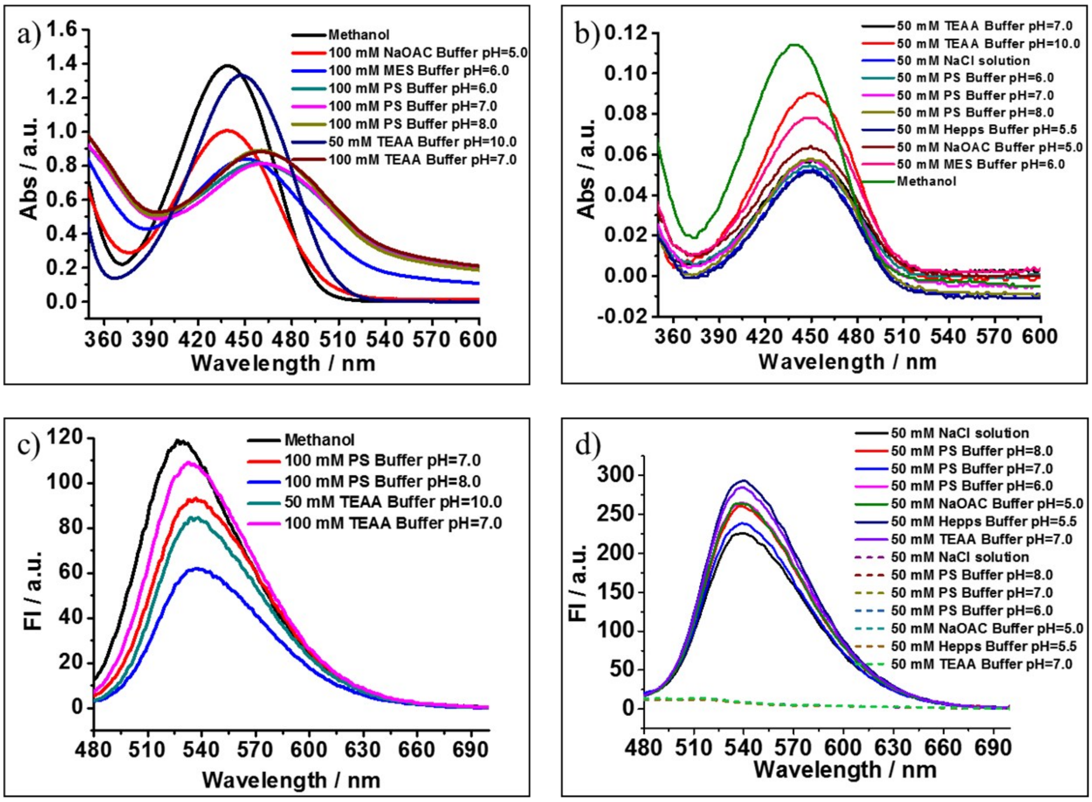

Figure S15. a) UV absorption spectra of LysU in the different buffer solutions; b) UV absorption spectra of Bioyin-lysU in the different buffer solutions; c) fluorescent emission spectra of LysU in the different buffer solutions (λ_{ex}: 439 nm); d) fluorescent emission spectra of Biotin-lysU (1 μM) (solid line) and Biotin-lys (1 μM) (dash line) in the different buffer solutions (λ_{ex}: 439 nm).

8. Polyacrylamide gel electrophoresis analysis

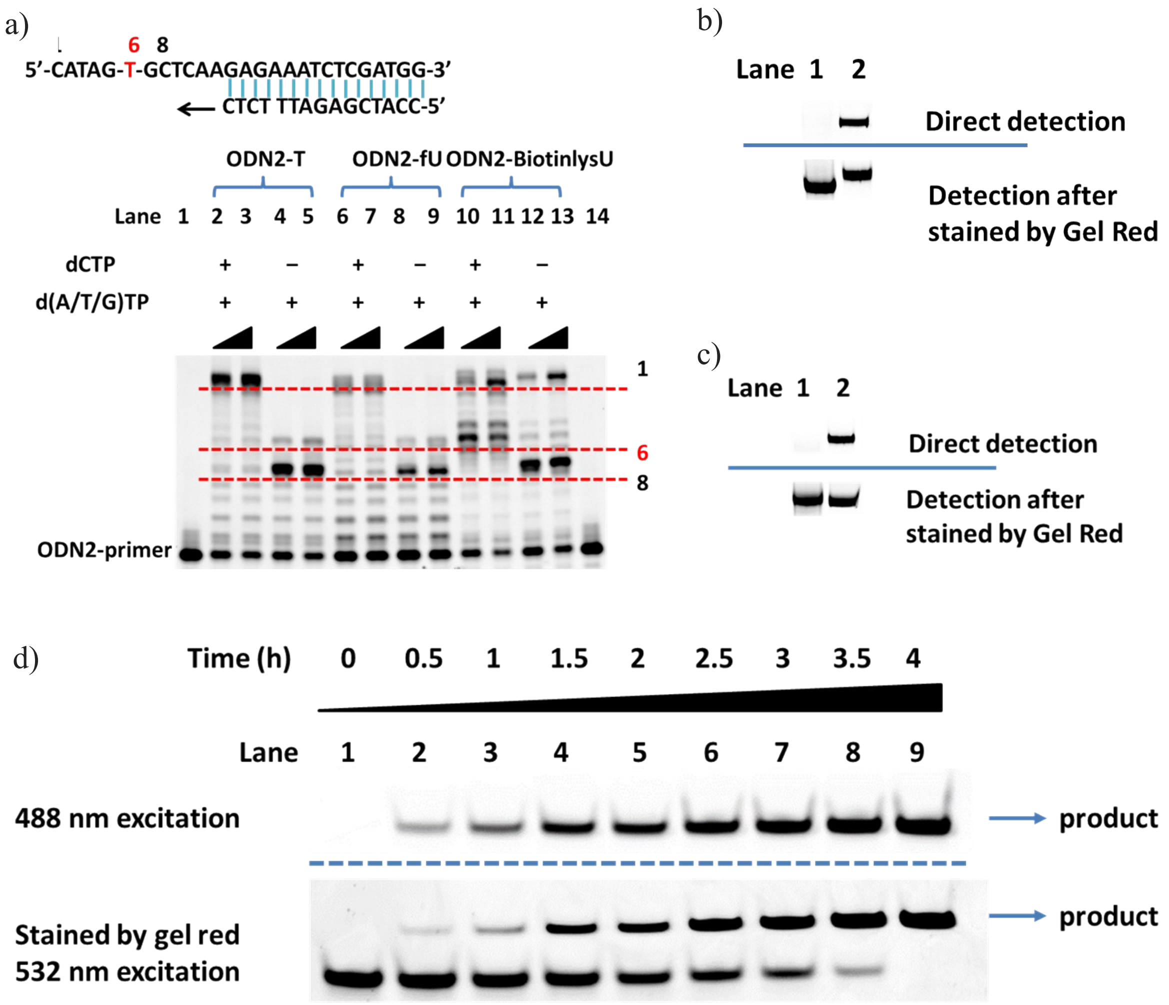

Figure S16. a) Primer-extension assay with Bsu DNA polymerase. Lane 1, 14: the FAM-labeled ODN2-primer marker; Lane 2, 3, 4, 5: unmodified DNA (ODN2-T); Lane 6, 7, 8, 9: ODN2-5fU; Lane 10, 11, 12, 13: ODN2-BiotinlysU (ODN2-5fU after incubation with Biotin-lys). Lane 2, 4, 6, 8, 10, 12, reaction time was 1 minute; Lane 3, 5, 7, 9, 11, 13, reaction time was 3 minutes. b) Denaturing polyacrylamide gel electrophoresis analysis of ODN-SS-fU and its canonical analogous ODN sequence (ODN-SS-T) which was treated with Biotin-lys (50 mM PS buffer, pH 7.0, 37°C, 6 h) before (above dash line, fluorescence mode, λ_{ex}: 488 nm) and after (below dash line, fluorescence mode, λ_{ex}: 532 nm) being stained with Gel Red. Lane 1: ODN-SS-T, lane 2: ODN-SS-fU. c) Denaturing polyacrylamide gel electrophoresis analysis of ds-DNA-fU and its canonical analogous ODN sequence (ds-DNA-T) which was treated with Biotin-lys (50 mM PS buffer, pH 7.0, 37°C, 6 h) before (above dash line, fluorescence mode, λ_{ex}: 488 nm) and after (below dash line, fluorescence mode, λ_{ex}: 532 nm) being stained with Gel Red. Lane 1: ds-DNA-T, lane 2: ds-DNA-fU. d) Denaturing polyacrylamide gel electrophoresis analysis of ODN-5fU treated with the reagent Biotin-lys (50 mM PS buffer, pH 7.0, 37°C) in different time (from 0 to 4 h) (above dash line, fluorescence mode, λ_{ex}: 488 nm) and after (below dash line, fluorescence mode, λ_{ex}: 532 nm) being

stained with Gel Red.

9. 5-formyluracil qPCR enrichment studies

Reaction procedure for enrichment of 5-formyluracil. Single stranded ODN (1 μg), Biotin-lys (50 mM, 1 μL) PS buffer (0.5 M, pH 7.0, 5 μL) were mixed and made up to a final volume of 50 μL at 37°C for 6 h in an Eppendorf tube in a thermomixer (Eppendorf, 1500 r.p.m.). The reactions were purified using mini quick spin oligo columns (Roche) pre-washed with water (2 × 300 μL) respectively. As for double stranded DNA, a mixture of ds-DNA-fU, ds-DNA-fC, ds-DNA-T (1 μg, respectively), 3M NaOH (1 μL) were made up to a final volume of 10 μL. After 42°C, 30 min incubaton, Biotin-lys (50 mM, 1 μL) PS buffer (0.5 M, pH 7.0, 5 μL) were added and made up to a final volume of 50 μL at 37°C for 6 h in an Eppendorf tube in a thermomixer (Eppendorf, 1500 r.p.m.). The mixture was also purified using mini quick spin oligo columns (Roche).

Pull down procedure. The pull down procedure was used with some modifications in a reported assay.[4] Streptavidin M280 Dynabead slurry (20 μL, Invitrogen) was washed with 200 μL 1x Binding & Wash buffer (5 mM Tris pH 7.5, 0.5 mM EDTA, 1 M NaCl, 0.01% Tween 20) three times. Then, it was diluted with 20 μL 2x Binding & Wash buffer. Input DNA (1 ng/ODN) and calf thymus (10 μg, sonicated into 100-200 bp, Sigama) were mixed and made up to a final volume of 20 μL and then added to the magnetic beads, before incubation for 15 minutes at 25°C on a nutator. After that, beads were washed three times with 200 μL of 1x Binding & Wash buffer. DNA was eluted by treated with 100 μL Elution Buffer (95% formamide, 10 mM EDTA, pH 8.2) at 90°C for 5 min. Magnetic beads were collected using a magnet (Promega) and the eluant was saved and immediately placed on ice. The beads were again incubated with 50 μL Elution Buffer for 5 min to elute any residual DNA. The chilled DNA was once again placed on the magnet to remove any residual beads. The eluant was added with 200 μL chilled water, 28 μL 3 M NaCl, 2 μg glycogen (Invitrogen) and 900 μL chilled 100% ethanol. The DNA was precipitated by incubating this mixture for 1 h at -80°C, then centrifuging at 14000 g for 30 min at 4°C. The precipitated DNA was washed three times with 900 μL chilled 70% ethanol, all ethanol was removed by a centrifugation at 14000 g for 5 min and subsequent aspiration, and the DNA was dried by Speedvac (Labconco Corporation, USA), then redissolved in 25 μL ddH$_2$O for further qPCR analysis.

qPCR analysis. qPCRs were performed using a CFX-96 RealTime System (Bio-Rad, USA). Enriched DNA (1 μL), forward primer, reverse primer (1 μM, respectively, See Table of oligonucleotides sequences), 2x Hieff™ qPCR SYBR® Green Master Mix (5 μL, YEASEN, Shanghai, China) were mixed up to 10 μL volume. The mixture was subject to qPCR using protocol outlined by the manufacturer. DNA concentration was quantified by comparison with calibration lines of known concentration of input ODNs below.

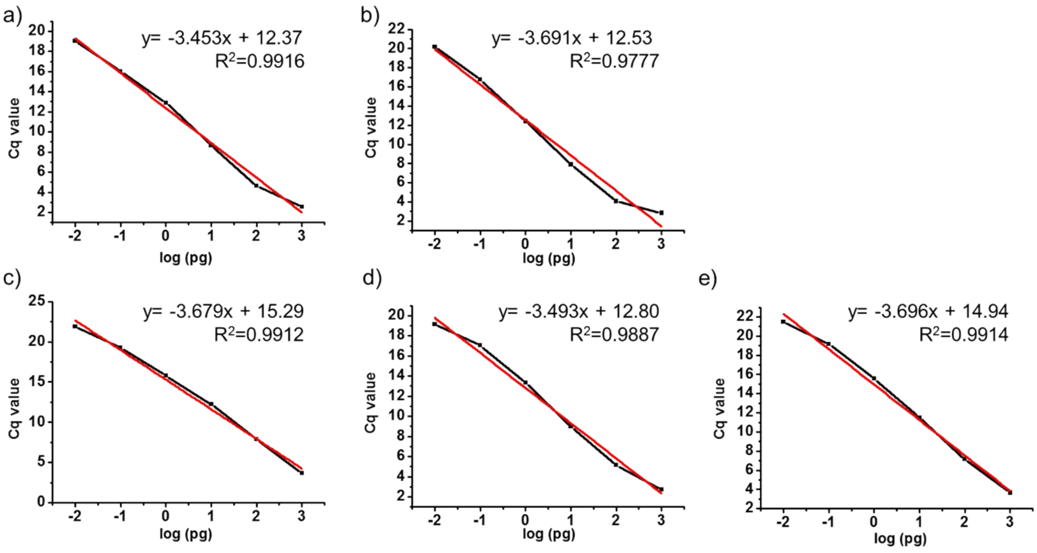

Figure S17. a) Example calibration line for ODN-SS-fU and ODN-SS-T; b) Example calibration line for ODN-SS-fC and ODN-SS-C c) Example calibration line for ds-DNA-fU; d) Example calibration line for ds-DNA-fC; e) Example calibration line for ds-DNA-T.

10. References

(1) C. Liu, Y. Chen, Y. Wang, F. Wu, X. Zhang, W. Yang, J. Wang, Y. Chen, Z. He, G. Zou, S. Wang, X. Zhou. *Nano Res*., 2017, DOI: 10.1007/s12274-017-1445-2.

(2) H. Yu, Y. Xiao, L. Jin, *J. Am. Chem. Soc.*, 2012, **134**, 17486.

(3) P. Guo, X. Xu, X. Qiu, Y. Zhou, S. Yan, C. Wang, C. Lu, W. Ma, X. Weng, X. Zhang, X. Zhou, *Org. Biomol. Chem.* 2013, **11**, 1610.

(4) W. A. Pastor, U. J. Pape, Y. Huang, H. R. Henderson, R. Lister, M. Ko, E. M. McLoughlin, Y. Brudno, S. Mahapatra, P. Kapranov, M. Tahiliani, G. Q. Daley, X. S. Liu, J. R. Ecker, P. M. Milos, S. Agarwal, A. Rao, *Nature*, 2011, **473**, 394.
